# Osteomimicry of Mammary Adenocarcinoma Cells *In Vitro*; Increased Expression of Bone Matrix Proteins and Proliferation within a 3D Collagen Environment

**DOI:** 10.1371/journal.pone.0041679

**Published:** 2012-07-24

**Authors:** Rachel F. Cox, Allan Jenkinson, Kerstin Pohl, Fergal J. O’Brien, Maria P. Morgan

**Affiliations:** 1 Department of Molecular and Cellular Therapeutics, Royal College of Surgeons in Ireland, Dublin, Ireland; 2 Anatomy Department, Royal College of Surgeons in Ireland, Dublin, Ireland; 3 Trinity Centre for Bioengineering, Trinity College Dublin, Dublin, Ireland; Institut de Génomique Fonctionnelle de Lyon, France

## Abstract

Bone is the most common site of metastasis for breast cancer, however the reasons for this remain unclear. We hypothesise that under certain conditions mammary cells possess osteomimetic capabilities that may allow them to adapt to, and flourish within, the bone microenvironment. Mammary cells are known to calcify within breast tissue and we have recently reported a novel *in vitro* model of mammary mineralization using murine mammary adenocarcinoma 4T1 cells. In this study, the osteomimetic properties of the mammary adenocarcinoma cell line and the conditions required to induce mineralization were characterized extensively. It was found that exogenous organic phosphate and inorganic phosphate induce mineralization in a dose dependent manner in 4T1 cells. Ascorbic acid and dexamethasone alone have no effect. 4T1 cells also show enhanced mineralization in response to bone morphogenetic protein 2 in the presence of phosphate supplemented media. The expression of several bone matrix proteins were monitored throughout the process of mineralization and increased expression of collagen type 1 and bone sialoprotein were detected, as determined by real-time RT-PCR. In addition, we have shown for the first time that 3D collagen glycosaminoglycan scaffolds, bioengineered to represent the bone microenvironment, are capable of supporting the growth and mineralization of 4T1 adenocarcinoma cells. These 3D scaffolds represent a novel model system for the study of mammary mineralization and bone metastasis. This work demonstrates that mammary cells are capable of osteomimicry, which may ultimately contribute to their ability to preferentially metastasize to, survive within and colonize the bone microenvironment.

## Introduction

Bone is one of the most preferential metastatic target sites for breast cancers [1], although the precise molecular mechanisms underlying this preference have yet to be elucidated. Mammary cells are known to mineralize, giving rise to mammographic microcalcifications, which are routinely used for the early detection of breast cancer. Up to 50% of all nonpalpable breast cancers and up to 90% of ductal carcinoma *in situ* (DCIS) are detected through mammographic microcalcifications [2], [3]. On a molecular level, there are two distinct forms of mammary microcalcifications; calcium oxalate and hydroxyapatite [4]. Calcium oxalate is mostly associated with benign breast lesions, whereas hydroxyapatite is associated with both benign and malignant breast tumors [5], [6], [7]. Hydroxyapatite is also a well documented component of bone, the deposition of which in bone tissue requires the coordinated expression of several bone matrix proteins, synthesized by cells of the osteoblastic lineage [8].

The functional role of hydroxyapatite deposition within the breast tumor microenvironment has been largely overlooked in the literature. However, we have previously shown that exogenous hydroxyapatite enhances the mitogenesis of mammary cell lines *in vitro* [9], suggesting that the presence of hydroxyapatite calcifications could potentially aggravate tumor growth. We have also demonstrated that hydroxyapatite upregulates the production of matrix metalloproteinases (MMPs) in mammary cell lines [9]. MMPs are well known to be involved in the degradation of the basement membrane, facilitating cancer cells metastasizing to surrounding tissues [10]. More recently, we have shown that hydroxyapatite enhances migration of a metastatic mammary cell line, whereas calcium oxalate has no effect [11], again suggesting that hydroxyapatite deposition may contribute to the metastatic process. We have also recently demonstrated that invasive mammary cell lines are capable of producing hydroxyapatite *in vitro* when exposed to an osteogenic cocktail [11]. A mechanism for mammary cell mineralization was proposed which centered on an imbalance between the enhancers and inhibitors of physiological mineralization [11]. Other studies have reported an overexpression of several bone matrix proteins, including bone sialoprotein, osteopontin and osteonectin, in breast cancer biopsies containing microcalcifications [12], [13].

We hypothesise that osteomimicry may represent an overlooked property of breast cancer cells that could contribute to the metastatic process by ensuring the cancer cells are primed to survive within the bone microenvironment. In this study we identify the components within the osteogenic cocktail essential for mineralization and we investigate whether mammary cells, which are capable of depositing hydroxyapatite, do so in a manner similar to osteoblasts. In addition, we examine the potential of 3D collagen scaffolds, engineered to represent the bone microenvironment, as a model for bone metastasis.

## Results

### β-glycerophosphate Alone is Sufficient to Induce Mineralization of 4T1 Cells

**Figure 1:**
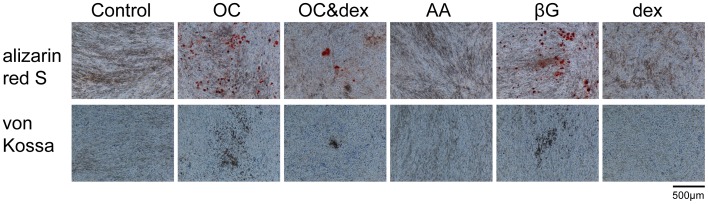
Investigating the effect of the individual components of the osteogenic cocktail on 4T1 cell mineralization. Alizarin red S and von Kossa staining are positive for calcium (red) and calcium phosphate (black/brown) respectively in the OC, OC&dex and βG groups on day 14. No positive staining was observed in the control, AA or dex groups. Representative images were taken at 100× magnification (n = 3) and the scale bar represents 500 µm. Control = regular growth media. AA = 50 µg/ml ascorbic acid. βG = 10 mM β-glycerophosphate. OC (osteogenic cocktail) = 50 µg/ml AA and 10 mM βG. Dex = 100 nM dexamethasone.

We have previously established that the metastatic mouse mammary 4T1 cell line is capable of mineralizing in the presence of an osteogenic cocktail, which consists of ascorbic acid and β-glycerophosphate with or without dexamethasone. A typical mineralizing nodule is shown in Figure S1 (Figure S1) in the supporting information. In this study the contribution of the individual components of the osteogenic cocktail used to induce mineralization was investigated. Positive staining for calcium (red) and calcium phosphate (black/brown) was observed with alizarin red S and von Kossa staining respectively after 14 days of treatment in the presence of 10 mM β-glycerophosphate alone, which was comparable to the staining observed in the osteogenic cocktail group (50 µg/ml ascorbic acid, 10 mM β-glycerophosphate ) at this time point (Figure 1). Positive staining was also detected in the osteogenic cocktail with dexamethasone group (50 µg/ml ascorbic acid, 10 mM β-glycerophosphate with 100 nM dexamethasone), but to a lesser extent than OC without dexamethasone. No positive staining was detected in response to treatment with ascorbic acid alone or dexamethasone alone, which was comparable to the control group grown in regular growth media.

### 4T1 Cells Mineralize in Response to β-glycerophosphate in a Dose Dependent Manner

**Figure 2:**
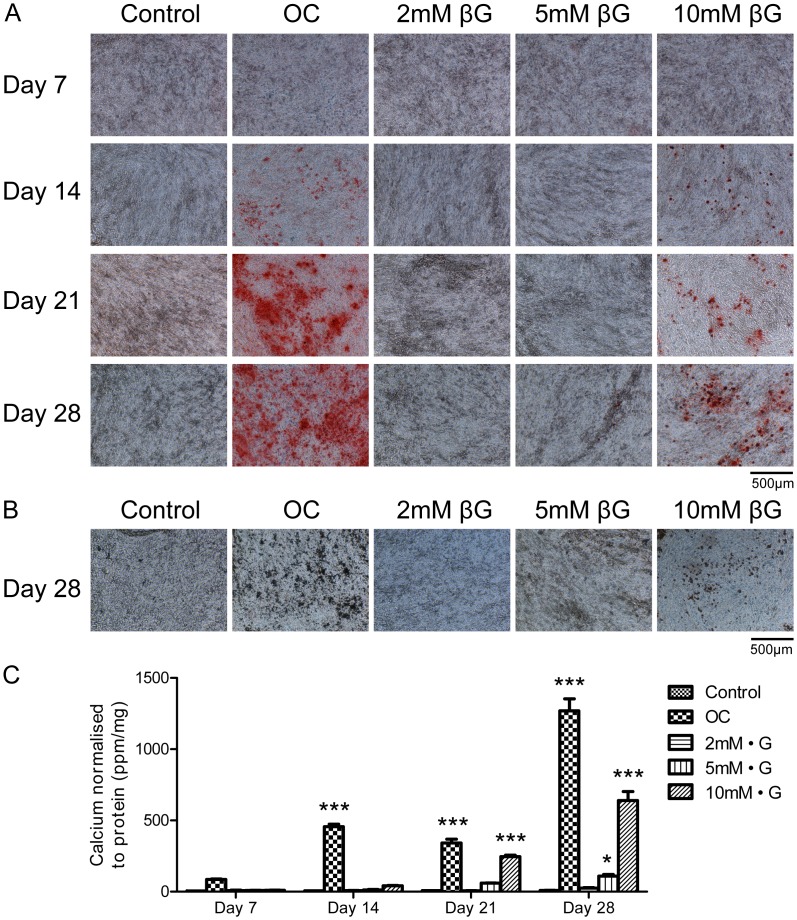
Investigating the effect of increasing concentrations of β-glycerophosphate on 4T1 cell mineralization. Representative images were captured at 100× magnification and the scale bars represent 500 µm. (A) Positive alizarin red S staining for calcium (red) was observed in the OC, 5 mM βG and 10 mM βG treated groups, beginning on days 14, 28 and 14 respectively. (B) Positive von Kossa staining for calcium phosphate (black/brown) was observed in the OC, 5 mM βG and 10 mM βG treated groups on day 28. (C) The calcium content of 4T1 cells as determined by the o-cresolphthalein calcium assay and normalized to protein. Increases in calcium were observed in the OC and 10 mM βG treated groups over time. Each point represents the mean amount of calcium measured in ppm normalized to protein measured in mg, +/− SEM, n = 3, two-way ANOVA. *P<0.05, ***P<0.001 vs. control at each time point. OC (osteogenic cocktail) = 50 µg/ml ascorbic acid and 10 mM β-glycerophosphate. βG = β-glycerophosphate.

4T1 cells were grown in culture plates for 28 days in the presence of regular growth media (control), the osteogenic cocktail (OC; 10 mM β-glycerophosphate and 50 µg/ml ascorbic acid), and increasing concentrations of β-glycerophosphate (βG; 2 mM, 5 mM and 10 mM). Positive staining for calcium was detected in the 10 mM βG group beginning on day 14 using alizarin red S (Figure 2A), with the intensity of the stain increasing over time up to day 28. Faint positive staining was detected in the 5 mM and 2 mM βG groups by day 28. These results were also confirmed using von Kossa staining, as shown by the day 28 representative images (Figure 2B). Positive staining for calcium phosphate (black/brown) was observed in the 5 mM and 10 mM βG group by this time point. A calcium assay was also used to quantify the results (Figure 2C). The greatest increase in calcium levels over time was observed in the OC group (P<0.001 vs. control on days 14, 21 and 28). In addition, by day 28 an 80-fold increase in calcium levels was detected in the 10 mM βG group, a 14-fold was observed in the 5 mM βG group and a 3.5-fold increase was detected in the 2 mM βG group, indicating a dose response. No changes in calcium normalized to protein were detected in the control group over time.

### 4T1 Cells Mineralize in Response to Inorganic Phosphate in a Dose Dependent Manner

**Figure 3:**
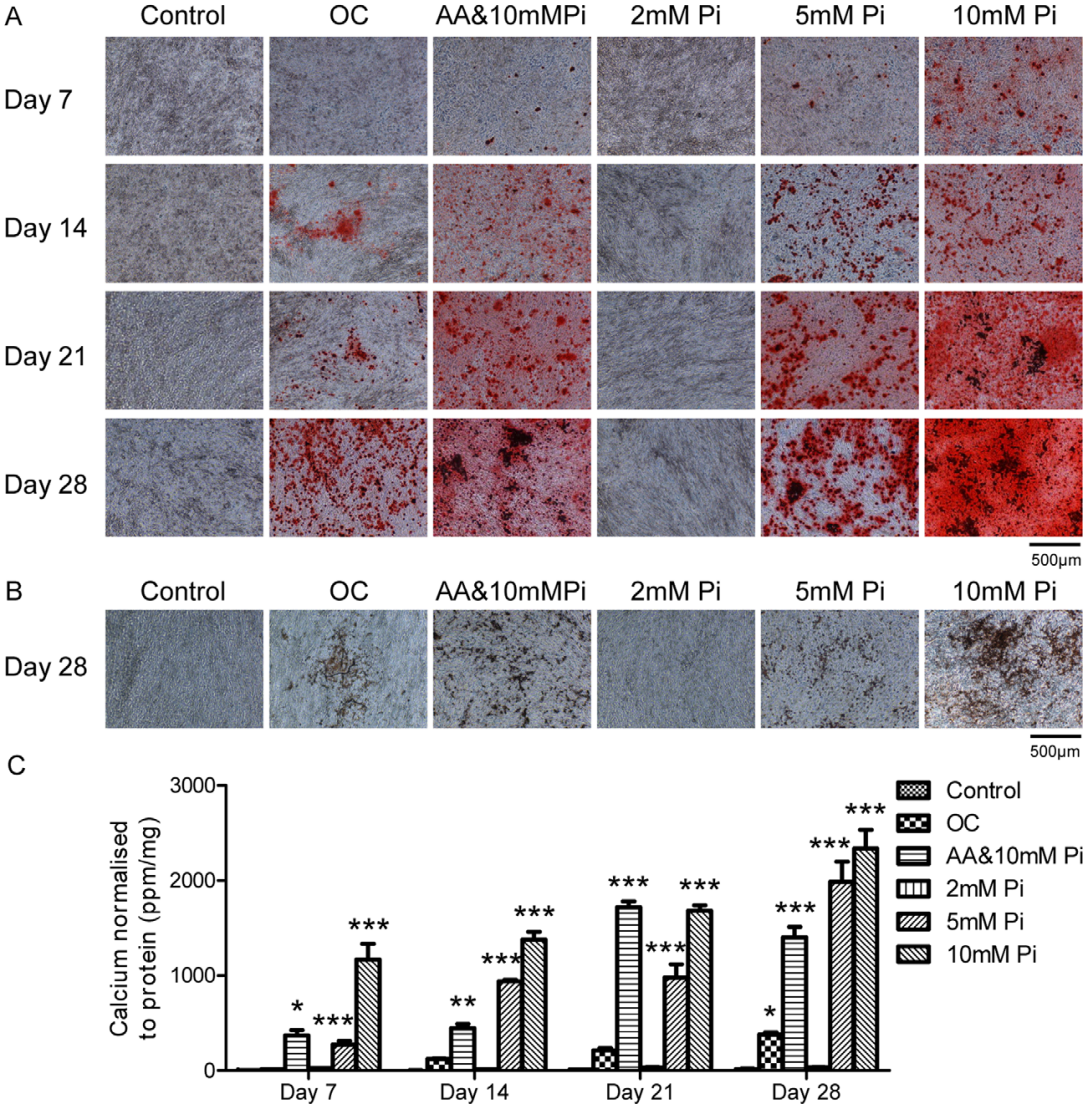
Investigating the effect of increasing concentrations of inorganic phosphate on 4T1 cell mineralization. Representative images were captured at 100× magnification and the scale bars represent 500 µm. (A) Positive alizarin red S staining for calcium (red) was observed in the OC treated group by day 14 and on day 7 for the AA&10 mM Pi, 5 mM Pi and 10 mM Pi treated groups. (B) Positive von Kossa staining for calcium phosphate (black/brown) was observed in the OC, AA&10 mM Pi, 5 mM Pi and 10 mM Pi on day 28. (C) The calcium content of 4T1 cells as determined by the o-cresolphthalein calcium assay normalized to protein. Increases in calcium were observed in the OC, AA&10 mM Pi, 5 mM Pi and 10 mM Pi groups over time, with the greatest increase detected in the 10 mM Pi group. Each point represents the mean amount of calcium measured in ppm normalized to protein measured in mg, +/− SEM, n = 3, two-way ANOVA. *P<0.05, **P<0.01, ***P<0.001 vs. control at each time point. OC (osteogenic cocktail) = 50 µg/ml ascorbic acid and 10 mM β-glycerophosphate. AA = 50 µg/ml ascorbic acid. Pi = inorganic phosphate.

Having established that the 4T1 cells are capable of mineralizing in the presence of organic phosphate, the effect of inorganic phosphate was also investigated. The 4T1 cells were grown in culture plates for 28 days in the presence of regular growth media (control), the osteogenic cocktail (OC; 10 mM β-glycerophosphate and 50 µg/ml ascorbic acid), AA&10 mM Pi (10 mM inorganic phosphate and 50 µg/ml ascorbic acid) and increasing concentrations of inorganic phosphate alone (Pi; 2 mM, 5 mM and 10 mM). The 4T1 cells began to stain positive for calcium (red; alizarin red S staining) on day 7 when treated with AA&10 mM Pi, 5 mM Pi alone or 10 mM Pi alone (Figure 3A). This staining increased in intensity over time, with the strongest staining in the 10 mM Pi group. Faint positive staining was observed in the 2 mM Pi group by day 28. A similar delayed pattern of staining for calcium phosphate (black/brown) was also observed as shown by representative day 28 von Kossa images (Figure 3B). Positive staining was observed in the OC, AA&10 mM Pi, 5 mM Pi and 10 mM Pi, with the strongest staining again in the 10 mM Pi group. A calcium assay was used to quantify the increase in cellular calcium (Figure 3C). Increases in calcium were detected for the OC group over time (P<0.05 day 28 vs. control group). However, the calcium levels for the AA&10 mM Pi, 5 mM Pi and 10 mM Pi groups were all consistently higher than the OC group at each time point and were elevated from day 7 onwards. The largest increase in calcium levels were detected in the 10 mM Pi group on day 28 (P<0.001 vs. control group).

### BMP2 Enhances Mineralization of 4T1 Cells in the Presence of the Osteogenic Cocktail

**Figure 4:**
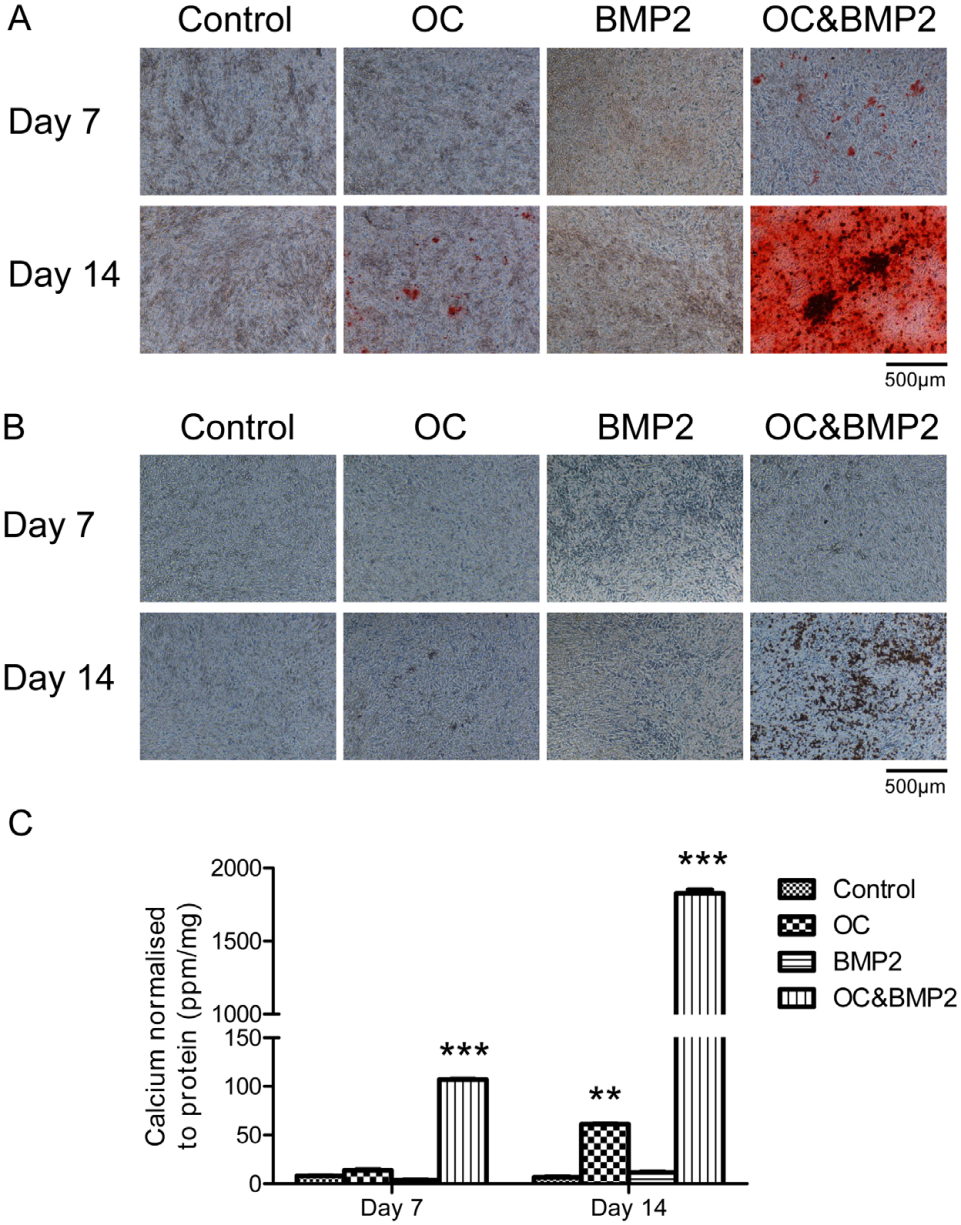
The effect of exogenous BMP2 on 4T1 cell mineralization. Representative images were captured at 100× magnification and the scale bars represent 500 µm. Positive staining for calcium (red) and calcium phosphate (black/brown) were observed in the OC&BMP2 group by day 7 and increased in intensity by day 14, as shown by alizarin red S (A) and von Kossa (B) staining. Positive staining was detected in the OC group by day 14 using both alizarin red S and von Kossa stains. (C) The calcium content of 4T1 cells as determined by the o-cresolphthalein calcium assay normalized to protein. By day 14 the calcium levels of the OC&BMP2 group are 30-fold greater than the OC group. Each point represents the mean amount of calcium measured in ppm normalized to protein measured in mg, +/− SEM, n = 3, two-way ANOVA. **P<0.01 OC vs. control on day 14. ***P<0.001 OC&BMP2 vs. all other groups on days 7 and 14. OC (osteogenic cocktail) = 50 µg/ml ascorbic acid and 10 mM β-glycerophosphate. BMP2 = 100 ng/ml human recombinant bone morphogenetic protein 2.

4T1 cells were seeded into culture plates and grown for up to 14 days in the presence of regular growth media (control), the osteogenic cocktail (OC; 50 µg/ml ascorbic acid and 10 mM β-glycerophosphate in regular growth media), human recombinant bone morphogenetic protein 2 (BMP2: 100 ng/ml BMP2 in regular growth media) or BMP2 in combination with the osteogenic cocktail (OC&BMP2). No positive staining for calcium (red) or calcium phosphate (black/brown) was detected using alizarin red S and von Kossa staining respectively, in the control group or the BMP2 alone group for up to 14 days (Figures 4A and 4B). Positive alizarin red S and von Kossa staining was observed in the OC treated group by day 14. Positive alizarin red S and von Kossa staining was detected in the OC&BMP2 group on day 7 and the intensity of the staining increased by day 14. These results were confirmed by a quantitative calcium assay (Figure 4C). An increase in calcium levels were detected in the OC group on day 14 compared to the control group (P<0.01), however these changes are overshadowed by the OC&BMP2 group at this time point, which is ∼30-fold greater than the OC group (P<0.001).

### Expression of Bone Matrix Proteins during 4T1 Mammary Cell Mineralization

**Figure 5:**
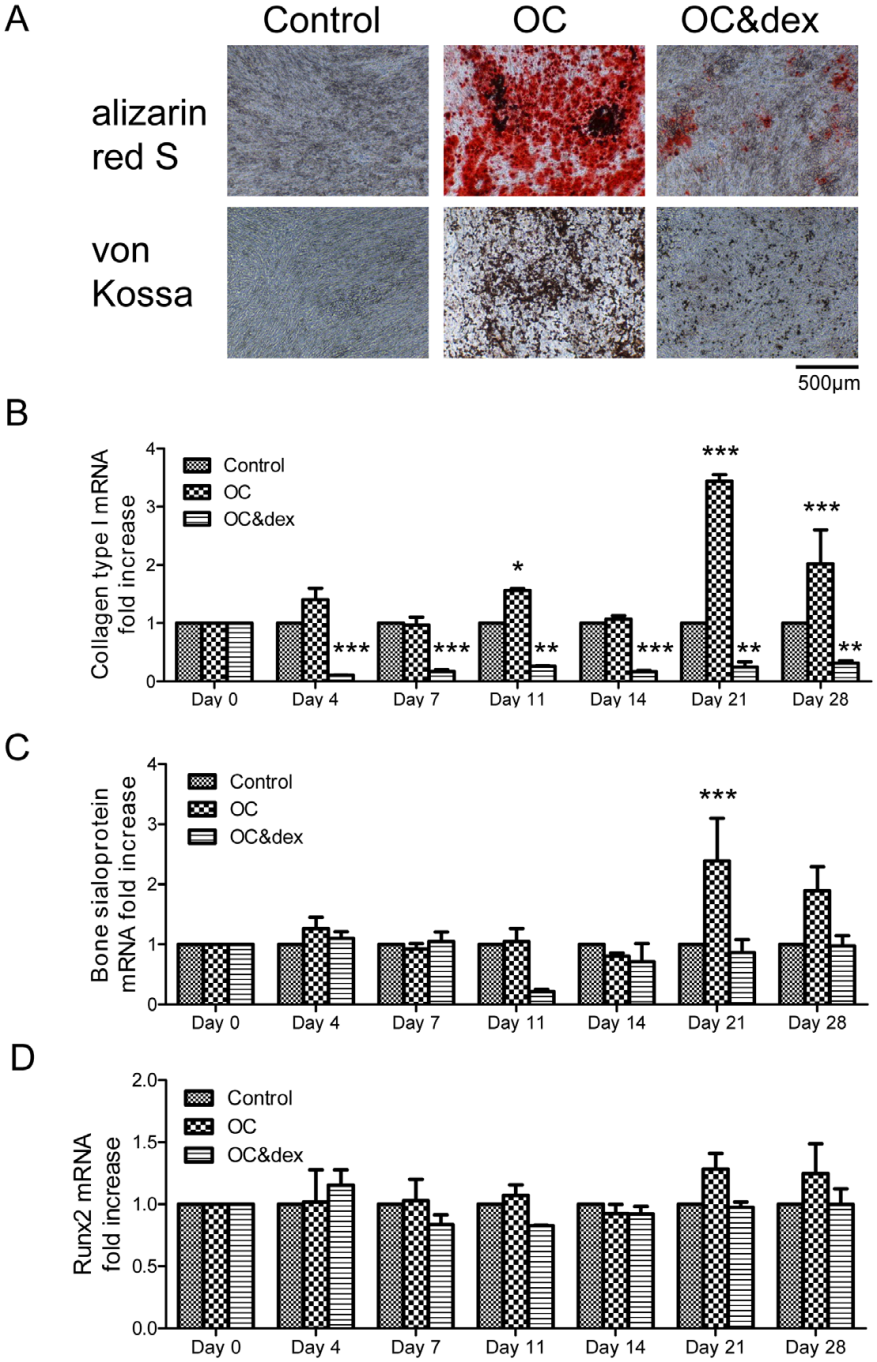
Investigating the expression of several bone markers in mineralizing 4T1 cells using real-time RT-PCR. (A) Alizarin red S and von Kossa staining of 4T1 cells after 28 days as viewed under a light microscope at 100× magnification (n = 3). The scale bar represents 500 µm. Positive staining was observed for calcium (red) and calcium phosphate (black/brown) using alizarin red S and von Kossa staining respectively, in the OC group and to a lesser extent in the OC&dex group. (B) There is a decrease in col1a1 (collagen type 1, alpha 1) mRNA expression in the OC&dex group compared to the control group from days 4–28. In contrast, there is increased expression of col1a1 in the OC group on days 11, 21 and 28. *P<0.05 OC vs. control on day 11. **P<0.01 OC&dex vs. control on days 11, 21 and 28. ***P<0.001 OC vs. control on days 21 and 28, also OC&dex vs. control on days 4, 7 and 14. (C) On day 21 there is an increase in the expression of bone sialoprotein (BSP) mRNA in the OC group compared to the control and OC&dex groups. ***P<0.001 OC vs. control and OC&dex groups on day 21. (D) No changes in the expression of Runx2 mRNA were detected between the different treatment groups over time. All real-time RT-PCR results are expressed in arbitrary units and normalized to the control samples at each time point. Each point represents the mean +/− SEM, n = 3, two-way ANOVA. OC (osteogenic cocktail) = 50 µg/ml ascorbic acid and 10 mM β-glycerophosphate. Dex = 100 nM dexamethasone.

4T1 cells were grown in culture plates and treated with regular growth media (control), the osteogenic cocktail (OC) or the osteogenic cocktail including 100 nM dexamethasone (OC&dex). Cell monolayers were stained on day 28 with alizarin red S and von Kossa to confirm calcium deposition. When 4T1 cells were grown in the OC for up to 28 days, a strong positive stain for calcium (red) and calcium phosphate (black/brown) was observed using alizarin red S and von Kossa staining respectively (Figure 5A). Positive staining for both alizarin red S and von Kossa was also detected in the OC&dex group, however this was to a much lesser extent than that observed for the OC group.

Using this established model of mammary mineralization, the expression of several bone markers were investigated using real-time RT-PCR from days 0–28. The 4T1 cells were found to express col1a1 (collagen type 1, alpha 1) mRNA and there was a general trend for upregulation of this bone marker in the OC group compared to the control group, which was statistically significant on days 11 (Figure 5B; P<0.05), 21 (P<0.001) and 28 (P<0.001). In contrast, there is a general trend for decreased expression of col1a1 in the OC&dex group compared to the control group, which was statistically significant at all time points from days 4–28 (P<0.01 on days 11, 21 and 28; P<0.001 on days 4, 7 and 14). The 4T1 cells were also found to express bone sialoprotein (BSP) mRNA. In the OC group, there is a statistically significant increase in BSP expression compared to the control group on day 21 (P<0.001; Figure 5C). There was also a 4.6-fold decreased in BSP mRNA expression in the OC&dex group on day 11 compared to the control group. While the 4T1 cells were also found to express Runx2 mRNA, the expression did not change over time between the different treatment groups (Figure 5D).

### Mineralization of 4T1 Cells in 3D Collagen Scaffolds

**Figure 6:**
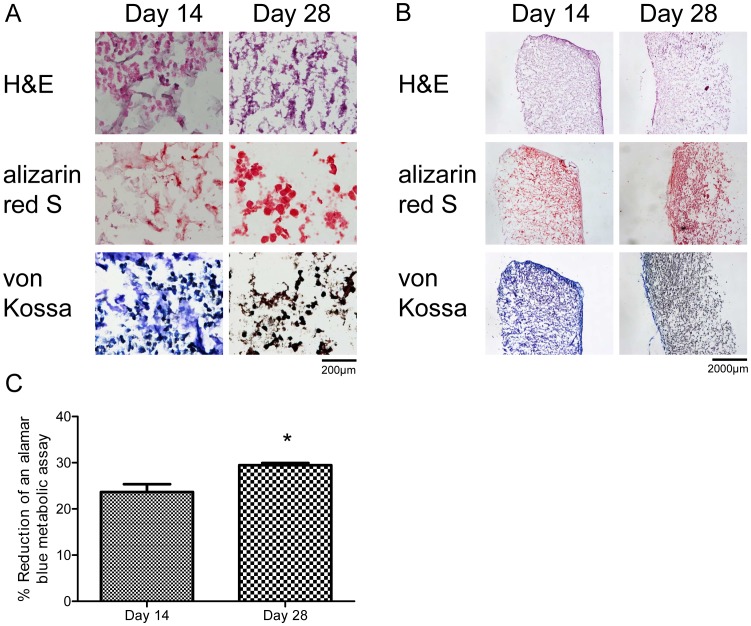
Assessing mineralization of 4T1 cells grown in an osteogenic cocktail within 3D collagen scaffolds. Representative images are shown at 400× and 40× magnifications and the scale bars represent 200 µm and 2000 µm respectively. (A) Positive hematoxylin and eosin (H&E) staining was observed at 400× magnification by day 14 and the intensity of the hematoxylin stain increased by day 28. Some positive alizarin red S staining for calcium (red) was detected by day 14. Complete positive staining with alizarin red S and von Kossa (including toluidine blue counterstain) was observed by day 28. (B) At 40× magnification the previously described patterns of staining are confirmed and minor contraction and disintegration of the scaffolds are observed over time. (C) Assessing cell viability of 4T1 cells grown within the 3D scaffolds using an alamar blue metabolic assay. An increase in cell viability was detected between day 14 and day 28. Each time point represent the mean % reduction in alamar blue +/− SEM, n = 3, students t-test. *P = 0.0284 day 28 vs. day 14. OC (osteogenic cocktail) = with 50 µg/ml ascorbic acid and 10 mM β-glycerophosphate.

To investigate tumor cell growth and osteomimicry at the bone metastatic site, the mineralization of the mouse mammary adenocarcinoma 4T1 cells were grown on collagen-glycosaminoglycan (GAG) scaffolds as the 3D structure more accurately represents the tumor-bone microenvironment. These highly porous scaffolds been engineered specifically for use in bone repair and possess an optimised pore structure and composition to facilitate adhesion and proliferation of osteoblasts [14], [15]. 4T1 cells were seeded into circular dehydrothermal-crosslinked collagen-GAG scaffolds and grown in the presence of the OC for up to 28 days. Positive H&E staining of 4T1 cells was observed in the center of the scaffolds by day 14 at 400× magnification (Figure 6A) confirming cell infiltration of the scaffold. At this time point, partial positive staining for calcium (red) and calcium phosphate (black/brown, counterstained with toluidine blue) were also detected using alizarin red S and von Kossa staining respectively (Figure 6A). By day 28, complete positive staining was observed for both alizarin red S and von Kossa staining. This pattern of staining is also observed at 40× magnification (Figure 6B) and in addition this magnification allows the effect of mineralization on the scaffold structure to be observed. Minor contraction of the scaffold appeared to take place over time from day 14 to day 28 and the collagen scaffolds appear to have disintegrated over time. Cell viability was also monitored using an alamar blue metabolic assay (Figure 6C) and was found to significantly increase from day 14 (22.2% reduced alamar blue) to day 28 (29.5% reduced alamar blue; P = 0.0284).

## Discussion

Mammographic microcalcifications are used for the early detection of breast cancer in its nonpalpable form. Several studies have shown that microcalcifications may also represent an important diagnostic indicator. In particular, mammary microcalcifications composed of hydroxyapatite are associated with both benign and malignant breast tumors, whereas calcium oxalate tends to be associated with benign lesions of the breast [5], [6], [7]. However, despite the diagnostic and potential prognostic value of microcalcifications, the mechanisms underlying their formation and their functional role in breast cancer progression remain unclear. In order to study this process, we have previously established an *in vitro* model of mammary cell mineralization using the metastatic 4T1 adenocarcinoma mammary cell line. These cells are capable of depositing hydroxyapatite after treatment with an osteogenic cocktail (OC) containing ascorbic acid and β-glycerophosphate with or without dexamethasone [11]. We hypothesised that under certain conditions mammary cells possess osteomimetic capabilities that may allow them to adapt to, and flourish within, the bone microenvironment.

In the current study, the role of the individual components of the OC were systematically investigated. It was found that ascorbic acid alone and dexamethasone alone do not result in 4T1 cell mineralization. Although dexamethasone is a common addition to osteogenic cocktails in osteogenic studies, the negative results of the dexamethasone treatment were not surprising, as dexamethasone has been shown to suppress mineralization of mouse osteoblast cells [16]. Treatment with 10 mM β-glycerophosphate alone was sufficient to induce mineralization after 14 days. The effect of β-glycerophosphate was found to be dose dependent as mineralization of the 4T1 cell line was enhanced with increasing concentrations of β-glycerophosphate using 2 mM, 5 mM and 10 mM. In addition, by day 28 it was clear that while 10 mM β-glycerophosphate alone was sufficient to induce mineralization, mineralization took place to a greater extent when ascorbic acid was also included. Therefore while ascorbic acid alone does not induce mineralization, its addition to the OC enhances the process. Ascorbic acid is known to upregulate the production of alkaline phosphatase [17], [18], which is a well documented enhancer of physiological mineralization [8].

In addition to using organic phosphate to induce mineralization of the mammary cells, the effect of inorganic phosphate, an essential and abundant physiological form of phosphate, was also investigated. Substituting the 10 mM organic β-glycerophosphate in the OC with 10 mM inorganic phosphate also resulted in 4T1 cell mineralization. The effect of inorganic phosphate was dose dependent, as mineralization was enhanced with increasing concentrations. In addition, mineralization was observed at earlier time points when using inorganic phosphate compared to organic phosphate. The comparative delay is likely due to hydrolysis of β-glycerophosphate to inorganic phosphate, which is time dependent [19]. In contrast, adding inorganic phosphate allows phosphate ions to be immediately available for use by the cells for mineral formation following initial treatment.

Bone morphogenetic protein 2 (BMP2) is another reagent commonly used to enhance osteoblast mineralization *in vitro* [20], [21], [22], [23], [24]. BMP2 is also known to be expressed in human breast cancers [25], [26] and injection of recombinant BMP2 into growing rodent mammary tumors results in mammary microcalcifications [27]. Therefore, the hypothesis that BMP2 may enhance 4T1 cell mineralization was investigated. It was found that 100 ng/ml BMP2 alone did not enhance 4T1 mineralization *in vitro*. However, when 100 ng/ml BMP2 was used in combination with the OC, mineralization was 30-fold greater compared to the OC alone. It is likely that within the tumor microenvironment, mammary cells that express BMP2 could use any available source of phosphate to produce mammary microcalcifications. While the source of phosphate used to produce microcalcifications *in vivo* remains unexplored, phosphate is abundant in the human body. It is feasible that within the tumor microenvironment, highly proliferative cancer cells may produce a localized increase in phosphate, which could lead to mineral deposition.

In order to elucidate the molecular mechanism involved in mammary mineralization, an area that remains largely uninvestigated, the expression of several bone markers were characterized using real-time RT-PCR. It was found that collagen type 1 alpha 1 (col1a1) and bone sialoprotein (BSP) mRNA were upregulated on day 21 in the mineralizing OC treated 4T1 cells. Both col1a1 and BSP are well documented enhancers of physiological mineralization and increased expression has been reported in the literature during this process [8], [28]. BSP is thought to act by nucleating hydroxyapatite [29]. Therefore BSP may play a similar role during mammary mineralization, especially as elevated BSP has been documented in human breast cancers [30], [31], [32], particularly in tumors containing microcalcifications [12]. The role of collagen type 1 during bone formation is to form a network of fibres, which support the growth of hydroxyapatite crystals [33], [34], [35]. Collagen is also a major component of the extracellular matrix of breast tissue and therefore may play a similar role as a natural scaffolding to support crystal growth. Dysregulation of collagen has been documented for breast cancer, as malignant breast tumors have increased collagen type 1 mRNA expression compared to benign breast tissue [36]. However, this is the first evidence to suggest that collagen may be associated with mammary microcalcifications.

The pattern of bone marker expression reported here adds weight to our hypothesis that the 4T1 cells mineralize in an active, regulated manner using a mechanism similar to osteoblasts. However, no changes in the expression of Runx2 mRNA were detected in the mineralizing 4T1 cells. Runx2 is widely considered to be involved in physiological mineralization by upregulating the expression of several bone matrix proteins including col1a1 and OPN [37], [38]. While Runx2 is the most well documented transcription factor associated with physiological mineralization, many others have been reported including osterix, Runx3 and calcineurin [39], [40], [41]. Therefore future studies will focus on their expression and potential involvement in mammary mineralization.

Having established and characterized the *in vitro* model of 4T1 cell mineralization in monolayer, next we focused on further developing this model in order to study bone metastasis. This was achieved using 3D collagen scaffolds, which are highly porous engineered biomaterials that have been well documented as supporting the growth and mineralization of osteoblasts [15], [42], [43]. While engineered biomaterials were originally developed for direct clinical applications, this technology has become a powerful tool in other biomedical research areas, including cancer research [44]. Cell growth in 3D is more reminiscent of physiological growth, compared to cells grown in monolayer [44]. 3D models may help bridge the gap between traditional 2D monolayer cell culture methods and animal models [44]. The scaffolds used in this study provide a 3D array of collagen fibres that support hydroxyapatite crystal growth in a manner that mimics the bone microenvironment. It was shown here for the first time that the scaffolds are capable of supporting the growth of adenocarcinoma mammary cells, as the 4T1 cells were seen to fully infiltrate the scaffolds by day 14 as shown by H&E staining of OC treated samples. It was also found that the mammary 4T1 cells are capable of mineralizing within this 3D environment in a similar manner to that previously reported for the 4T1 cells grown in monolayer. Mineralization of the 4T1 cells began on day 14 in the 3D scaffolds and extensive mineralization was observed by day 28. It was also shown that cell viability was not compromised for up to 28 days and while minor contraction and disintegration of the scaffolds took place, this did not affect the process of mineralization. These bioengineered scaffolds represent a novel system for the study of mammary mineralization within the tumor-bone microenvironment.

In summary we have shown that mammary adenocarcinoma 4T1 cells are capable of osteomimicry. Exogenous sources of phosphate and BMP2 were found to enhance mineralization of the 4T1 cell line, which has been well documented in osteoblast cultures. In addition, we have shown that the bone matrix proteins col1a1 and BSP are differentially expressed during the process of mammary mineralization *in vitro*. Also for the first time, it was shown that mammary cells are capable of mineralizing within a 3D collagen scaffold. Through expression of bone marker proteins and their capacity for growth and infiltration within a highly collagenous setting, adenocarcinoma cells demonstrate their innate ability to adapt to the hydroxyapatite rich microenvironment of bone. We suggest that mammary mineralization is not simply a process of cellular degeneration as has previously been suggested, but an actively regulated osteomimetic process that may have functional consequences contributing to breast cancer metastasis to bone.

## Materials and Methods

### Cell Lines and Media

The murine mammary adenocarcinoma 4T1 cell line is available from ATCC [45]. This cell line was maintained in a regular growth media consisting of low glucose DMEM, 10% FBS and 1% penicillin/streptomycin. All cell culture reagents were purchased from Sigma-Aldrich (Ireland and Biosera, UK).

### Induction of Mineralization

4T1 cells were seeded into 6-well culture plates (day −1) at a density of 1.5×10^5^ cells/well and the following day (day 0) the cells were treated with regular growth media with/without the following: an osteogenic cocktail (50 µg/ml ascorbic acid, 10 mM β-glycerophosphate with or without 100 nM dexamethasone), 50 µg/ml ascorbic acid, 100 nM dexamethasone, or increasing concentrations of β-glycerophosphate or inorganic phosphate (2 mM, 5 mM and 10 mM). In addition, the effect of recombinant human bone morphogenetic protein 2 (BMP2; ebiosciences, Hatfield, UK) on 4T1 cell mineralization was also investigated by adding 100 ng/ml to regular growth media or media containing the osteogenic cocktail. Cells were maintained in an atmosphere of 37°C and 5% CO_2_ and half of the media was replenished every three days for up to 28 days.

For 3D studies, in-house engineered circular collagen-glycosaminoglycan (GAG) scaffolds with a diameter of 12.5 mm were seeded with 2×10^6^ 4T1 cells. These scaffolds are fabricated using a lyophilisation technique [46], [47] which results in a highly porous (>99%) structure. Following lyophilisation, the scaffolds were crosslinked using a dehydrothermal process [14] in a vacuum oven at a temperature of 105°C. This process improves the mechanical properties of the scaffolds and also sterilises them for cell culture. Scaffolds were maintained in regular growth media for 2 days and were then treated with media containing the osteogenic cocktail (50 µg/ml ascorbic acid and 10 mM β-glycerophosphate). Cells were maintained at 37°C and 5% CO_2_ and samples were taken for analysis at 14 and 28 days. The scaffolds were fixed in formalin (10%) for 30 min at room temperature and placed in a Leica ASP 300 tissue processor overnight. The scaffolds were then cut in half and embedded in wax either horizontally or vertically. Samples were sectioned (10 µm) using the Leica RM2255 microtome (Nussloch, Germany). The sections were mounted onto polysine coated slides (Fisher Scientific, Loughborough, UK) and kept at room temperature until histological staining was carried out as described below.

### Histological Staining

4T1 cell monolayers were fixed with 10% formalin for 30 min followed by staining with alizarin red S (2%, pH 4.4, 4 min). For von Kossa staining, fixed cells were incubated with silver nitrate (5%) for 1 hour under an electric lamp, which was followed by sodium thiosulphate (5%) treatment for 2 min. For paraffin embedded scaffolds, serial sections (10 µm) were deparaffinised using xylene and rehydrated through a series of ethanol washes prior to staining with alizarin red S, von Kossa (using toluidine blue counterstain) or hematoxylin and eosin (H&E). Samples were dehydrated, incubated with xylene (30 min) and mounted with DPX. Images were captured using the Nikon Eclipse TS100 inverted microscope and Nikon Digital Sight DS-L1 camera system (Amstelveen, The Netherlands).

### Alamar Blue

Cell viability was monitored for cells grown in 3D collagen scaffolds using an alamar blue® metabolic assay according to the manufacturer’s instructions (Invitrogen, California, USA). Briefly, cell cultures were incubated with Alamar blue® (10% in regular growth media) at 37°C for 2 hours. The absorbance of the media from each well was measured in triplicate at 540 nm and 620 nm in a spectrophotometer and analysis was carried out according to the manufacturer’s instructions.

### Quantitative Calcium Assay

Calcium was extracted by incubating 4T1 cell monolayers with nitric acid (1 M; 1 hr). The absorbance of the samples when reacted with o-cresolphthalein (0.1 mg/ml) and 2-amino-2-methyl-1-propanol (90 mg/ml) was read at 572 nm [48]. A BCA protein assay (Novagen, Darmstadt, Germany) was performed on duplicate samples. The cells were suspended in RIPA lysis buffer (1× PBS; 1% NP-40, 0.5% sodium deoxycholate, 0.1% SDS) containing 1% protease inhibitor cocktail (Sigma Aldrich, Arklow, Ireland), stored on ice for 1 hr with occasional vortexing and centrifuged at 12000 rpm for 20 min. The BCA protein assay was carried out on the supernatant according to the manufacturer’s instructions.

### Real-time RT-PCR

Total RNA was extracted from 4T1 cells using trizol® (Invitrogen, California, USA) and was reverse transcribed using a high-capacity cDNA reverse transcription kit (Applied Biosystems, California, USA). Real-time RT-PCR was carried out using the real-time PCR thermocycler (Applied Biosystems 7500) for col1a1 (QT00162204, Qiagen, Hilden, Germany), bone sialoprotein (QT00115304, Qiagen) and Runx2 (QT00102193, Qiagen), using 18 s (QT01036875, Qiagen) as an endogenous control. Samples were heated at 95°C for 15 min. This was followed by a second stage of 15 sec at 94°C, 30 sec at 55°C, and 45 sec at 72°C, which was repeated 40 times.

### Statistical Analysis

All statistical analysis was carried out using GraphPad Prism 5 software (La Jolla, CA, USA). When comparing two groups, students t-tests were used. When analyzing multiple treatments over multiple time points, a two-way ANOVA was used. Post-hoc analysis was carried out when a significant p value of less than 0.05 was detected.

## Supporting Information

Figure S1 Visualisation of nodule formation with alizarin red S staining of 4T1 cells (positive for calcium (red)) treated with the OC on day 11. Representative image was taken at 100× magnification (n = 3). OC (osteogenic cocktail) = 50 µg/ml ascorbic acid and 10 mM β-glycerophosphate.
(JPG)
