# Supplementary figures and images for: Osteomimicry of Mammary Adenocarcinoma Cells In Vitro; Increased Expression of Bone Matrix Proteins and Proliferation within a 3D Collagen Environment
Source: PLoS One. 2012 Jul 24;7(7):e41679. doi: 10.1371/journal.pone.0041679 (PMC3404045; doi:10.1371/journal.pone.0041679)

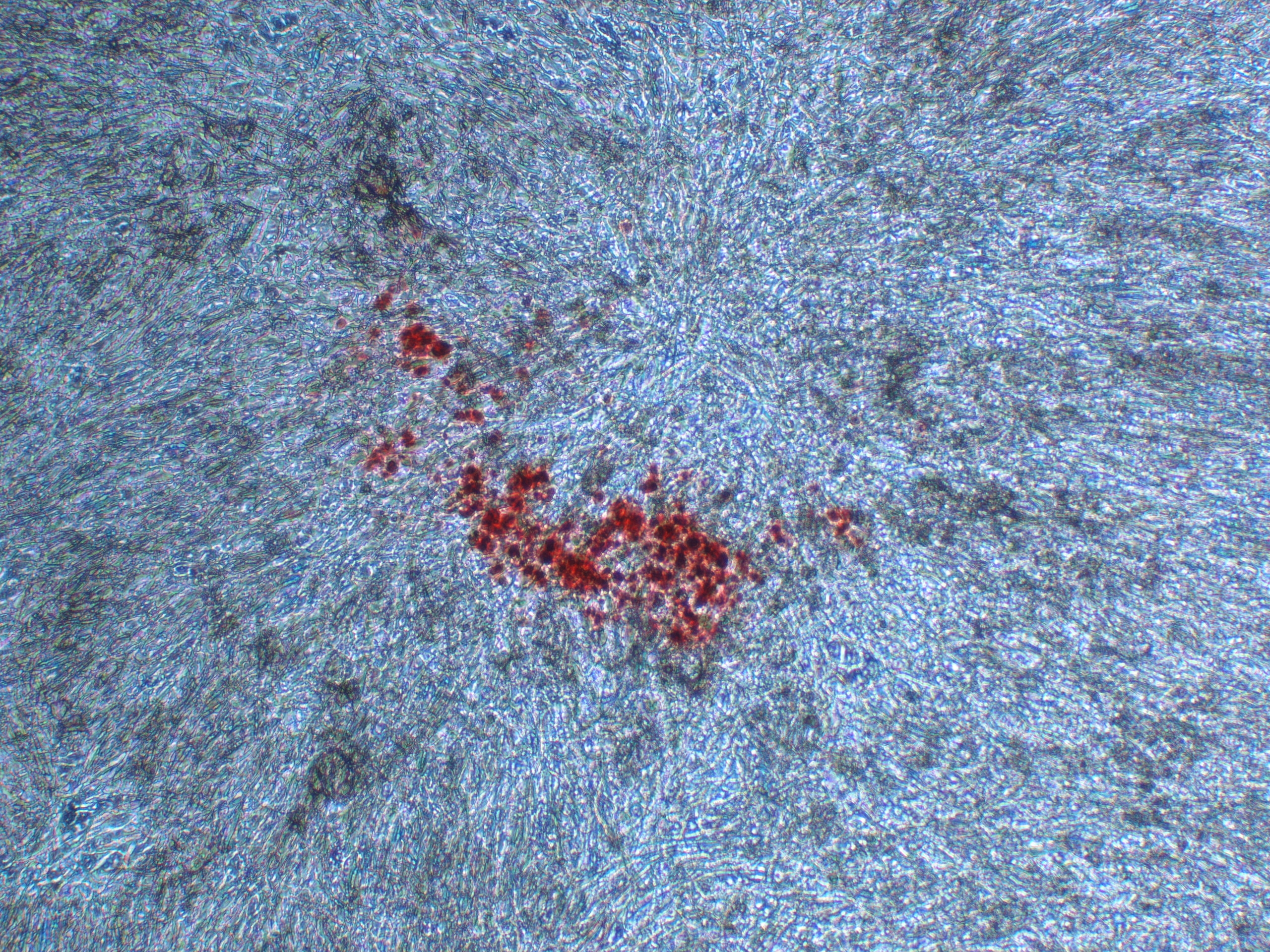

Supplement: Figure S1 — Visualisation of nodule formation with alizarin red S staining of 4T1 cells (positive for calcium (red)) treated with the OC on day 11. Representative image was taken at 100× magnification (n = 3). OC (osteogenic cocktail) = 50 µg/ml ascorbic acid and 10 mM β-glycerophosphate. (JPG) [file pone.0041679.s001.jpg]
